# Mechanical Properties of 3D-Printed Titanium Alloy Titanflex® Compared to Conventional Materials for Removable Denture Bases: An Experimental Study

**DOI:** 10.3390/ma18194563

**Published:** 2025-09-30

**Authors:** Ana Šango, Janoš Kodvanj, Petra Tariba Knežević, Davor Vučinić, Petra Besedić, Višnja Katić

**Affiliations:** 1 Department of Prosthodontics, Faculty of Dental Medicine, University of Rijeka, Krešimirova 40, 51000 Rijeka, Croatia; ana.sango@fdmri.uniri.hr (A.Š.); petra.tariba@fdmri.uniri.hr (P.T.K.); davor.vucinic@fdmri.uniri.hr (D.V.); 2 Department of Applied Mechanics, Faculty of Mechanical Engineering and Naval Architecture, University of Zagreb, Ivana Lučića 5, 10000 Zagreb, Croatia; janos.kodvanj@fsb.hr (J.K.); petra.besedic@fsb.hr (P.B.); 3 Clinical Hospital Centre Rijeka, Clinic of Dental Medicine, Krešimirova 42, 51000 Rijeka, Croatia; 4 Department of Orthodontics, Faculty of Dental Medicine, University of Rijeka, Krešimirova 40, 51000 Rijeka, Croatia

**Keywords:** dental prosthesis, three-dimensional printing, titanium, titanium alloys

## Abstract

This study investigates the mechanical properties of titanium (Titanflex®) and cobalt-chromium (Co-Cr) alloys for potential use in removable denture bases. Titanium alloys have gained attention due to their biocompatibility and regulatory concerns surrounding Co-Cr, which has been classified as a carcinogenic, mutagenic, and toxic to reproduction (CMR) substance under EU MDR (2017/745). Using selective laser melting (SLM), test specimens of Titanflex® and Co-Cr alloys were 3D-printed at different angles (0°, 45°, 90°) and compared to conventionally cast Co-Cr samples. Tensile testing was conducted to assess modulus of elasticity (E), proof stress (Rp_0.2_), ultimate tensile strength (Rm), elongation parameters (Ag, Agt, At), and maximum load (Fm). Results showed that Titanflex® printed at 45° (Ti45) exhibited the highest Rp_0.2_, Rm, and Fm, indicating superior strength and plastic resistance. Ti0 displayed the greatest elongation properties, highlighting titanium’s ductility. Co-Cr alloys demonstrated higher stiffness but lower ductility. Printing orientation significantly influenced mechanical properties, particularly in 3D-printed samples. Overall, Ti45 presented a balanced profile of strength and flexibility, making it a promising candidate for denture bases, while Co-Cr remains a rigid alternative with established clinical use. Future research should explore long-term performance under functional and biological conditions to guide clinical application.

## 1. Introduction

In the last decade, 3D printing has found increasingly widespread application in dental medicine due to its ability to precisely fabricate complex shapes, its rapid production capabilities, and its significant reduction in material consumption compared to casting and milling techniques [1]. This method generates no waste material, and the risk of human error is minimized. Titanium alloys have become increasingly popular in dental medicine due to their excellent mechanical properties, high biocompatibility, corrosion resistance, and non-magnetic nature [2]. The technology of 3D printing is being adapted to produce titanium implants as well as fixed and removable prosthetic restorations [1,3,4].

Existing research has demonstrated that titanium alloys offer numerous advantages compared to other materials and are increasingly used in maxillofacial and oral surgery, orthodontics, neurosurgery, and cardiovascular surgery [5,6]. A recent study by Deeban et al. (2024) investigated the mechanical properties of titanium alloy frameworks fabricated using selective laser melting (SLM) for complete dentures. The study found that SLM-produced titanium alloys exhibited high mechanical strength and precision, making them a viable alternative to traditional casting methods for dental prostheses [7]. Similarly, research by Kim et al. (2020) examined the mechanical and biological properties of 3D-printed titanium alloys and confirmed their suitability for dental applications [3]. 

Given the frequent hypersensitivity reactions observed in patients to cobalt-chromium (Co-Cr) alloys, titanium alloys provide a viable alternative due to their high biocompatibility. Sitalaksmi et al. (2019) highlighted a connection between COX-2 (cyclooxygenase-2)-induced T-cell accumulation and the development of allergies to chromium (Cr), further supporting the need for alternative materials like titanium alloys [8]. 

Since 2017, the regulatory framework of the European Union (EU) for Co-Cr alloys has undergone significant changes. The new EU Medical Device Regulation (MDR) (2017/745) classified cobalt (Co) as a carcinogenic, mutagenic, and toxic to reproduction (CMR) substance. The MDR came into effect in May 2021, and by May 2025, all medical devices must comply exclusively with the MDR approval process. The use of cobalt may be prohibited in certain medical devices containing more than 0.1% Co. Existing devices on the market will remain valid until 2024. Thus, during the 2021–2025 transition period, cobalt will be permitted for use in Co-Cr dental alloys under strict compliance with MDR (EU) 2017/745 and the CLP regulation (EC) No. 1272/2008 [9]. Patient documentation and warnings will also become mandatory. Titanium alloys are increasingly regarded as an effective replacement for Co-Cr alloys [10]. 

The mechanical properties of a material are crucial for its application as they determine its performance under various operational conditions. These properties are particularly significant in the design of dental prosthetic structures. When selecting a material, it is essential to consider all types of mechanical stresses and biological influences to which it will be exposed. The goal is to choose a material capable of withstanding all functional conditions without cracking, fracturing, or undergoing permanent plastic deformation, as such failures could render the denture bases nonfunctional [11]. Given that the biological and mechanical properties of metallic alloys are influenced by the manufacturing method (casting, rolling, forging, or 3D printing) and are subject to variation due to differences in crystal lattice structure, dental material manufacturers strive to develop alloys that meet the appropriate biomechanical ISO standards [3,12]. 

The proposed study focused on comparing the mechanical properties of the titanium alloy (Titanflex®) designed for 3D printing with Co-Cr dental alloys produced through 3D printing and casting for the fabrication of denture bases [13,14,15]. Of particular interest will be the impact of the 3D printing angle on the mechanical properties of Titanflex® and Co-Cr alloys, compared to cast Co-Cr alloy, which is considered the gold standard in clinical practice for fabricating denture bases. 

## 2. Materials and Methods

**Figure 1:**
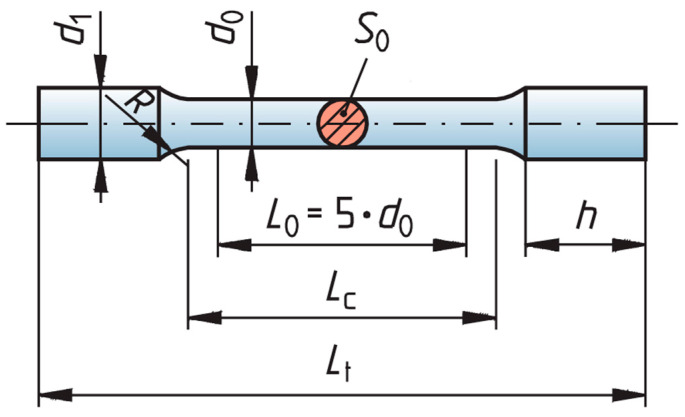
Cylindrical specimens design and dimensions. The dimensions of the test specimens used in the study were as follows: d_0_ = 4 mm, L_0_ = 20 mm, Lc_min_ = 24 mm, R_min_ = 3 mm, d_1_ = 5 mm, Lt_min_ = 60 mm, h_min_ = 16 mm, S_0_ = initial cross-section measured for every sample.

**Figure 2:**
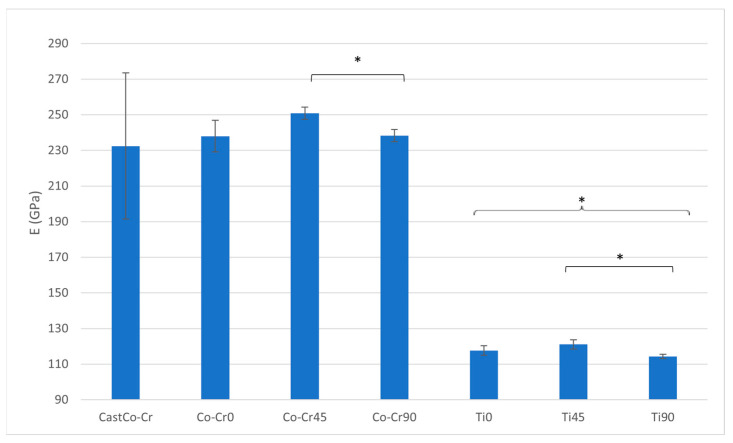
E ratio between tested materials. The graph shows the comparison of modulus of elasticity among cast Co-Cr alloy, 3D-printed Co-Cr alloy (Co-Cr0, Co-Cr45, Co-Cr90), and 3D-printed Titanflex® alloy (Ti0, Ti45, Ti90). Asterisks (*) indicate statistically significant differences (* *p* < 0.05). Error bars represent standard deviation.

The study utilized samples made from cast Co-Cr alloy, 3D-printed Co-Cr alloy and 3D-printed Titanflex® alloy. The Titanflex® alloy is produced from titanium powder, type 4, in accordance with EN ISO 22674 [16]. The powder has a particle size distribution of +10–45 μm. Its chemical composition consists of 89.4% titanium, 6.2% aluminum, and 4.0% vanadium, while the combined content of nitrogen, carbon, hydrogen, iron, and oxygen is below 0.4%. The Co-Cr alloy for 3D printing is produced in accordance with EN ISO 22674 for metallic materials used in fixed and removable restorations [16]. The powder has a particle size distribution of 15–45 μm. Its chemical composition is based predominantly on cobalt (approximately 60–65%) and chromium (about 25–30%), with minor additions of molybdenum, tungsten, silicon, manganese, and iron. The total content of carbon, nitrogen, and other trace elements remains below the maximum limits specified by the standard. The Co-Cr alloy used for casting is produced in accordance with EN ISO 22674 (Type 5) [16]. Its chemical composition consists of 62.5% cobalt, 29.5% chromium, 5.5% molybdenum, and 1.4% silicon, while the combined content of manganese, carbon, and nitrogen remains below 1%. The planned sample size was five for each alloy. The sample size was calculated using an online calculator with 80% power and a significance level of 0.05, based on data from the literature [17,18]. Samples were fabricated by casting biocompatible Co-Cr dental alloy and by 3D printing using the Trumpf TruPrint 1000 G06 (TRUMPF GmbH + Co. KG, Ditzingen, Germany) with biocompatible Co-Cr and Titanflex® dental alloys. The printed samples were produced in the form of uniform test specimens with standardized dimensions (Figure 1 and Figure 2). 

The 3D-printed samples were fabricated at three angles: 0°, 45°, and 90°, using selective laser melting (SLM) technology. Printing parameters, including temperature and scanning speed, were carefully controlled to ensure optimal sample quality [1,19,20]. Samples were produced according to a vector strategy, with printing parameters fully protected (Trumpf Strategy). In this context, selective laser melting (SLM) was performed using a powder particle size of 10–30 μm, a laser scanning speed of 600–1200 mm/s, and a laser power of 90–180 W, depending on the alloy composition. Both pulsed and continuous exposure modes were applied, selected according to the material and desired microstructural features, as these parameters are known to have a decisive influence on densification, microstructure, and the mechanical properties of the fabricated samples. All specimens were polished using the Dlyte polishing system (Wieland Dental, Penzberg, Germany) according to the manufacturer’s protocol. The procedure involved sequential polishing with diamond-impregnated brushes under controlled pressure and rotational speed, ensuring a standardized, high-gloss surface finish. The process was performed automatically to minimize operator variability and to achieve reproducible surface characteristics suitable for mechanical testing. Tensile tests were conducted using a Beta 50-5 testing machine (Messphysik, Feldkirchen, Austria) at the Laboratory for Experimental Mechanics, Faculty of Mechanical Engineering and Naval Architecture, University of Zagreb. The machine’s maximum load capacity was 50 kN. Static testing was performed in accordance with HRN EN ISO 6892-1:2019 [21], utilizing Method A1 (closed-loop strain rate control) with a traverse speed of 0.00025 mm/mm/s, as recommended for standard testing conditions. During testing, the following parameters were measured: E-Modulus (E), proof stress at 0.2% (Rp_0.2_), ultimate tensile strength (Rm), uniform elongation (Ag), total uniform elongation (Agt), breaking elongation (A), total breaking elongation (At), and maximum load (Fm). These measurements enabled a comprehensive analysis of the mechanical properties of the samples [3,21,22].

The statistical analysis was performed using a t-test for independent samples, with the significance level set at 0.05.

## 3. Results

The Titanflex® alloy (Ti) demonstrates a significantly lower modulus of elasticity (E) compared to all other materials (*p* < 0.05). Within the Titanflex® group, Titanflex® at 90° (Ti90) has a significantly lower E than Titanflex® at 45° (Ti45) (*p* < 0.05). The Co-Cr alloy at 90° (Co-Cr90) exhibits a significantly lower E compared to the Co-Cr alloy at 45° (Co-Cr45) (*p* < 0.05). The cast Co-Cr alloy (cast Co-Cr) shows intermediate values for E, but no statistical differences are noted between it and the other Co-Cr alloys (Figure 2 and Table 1).

**Figure 3:**
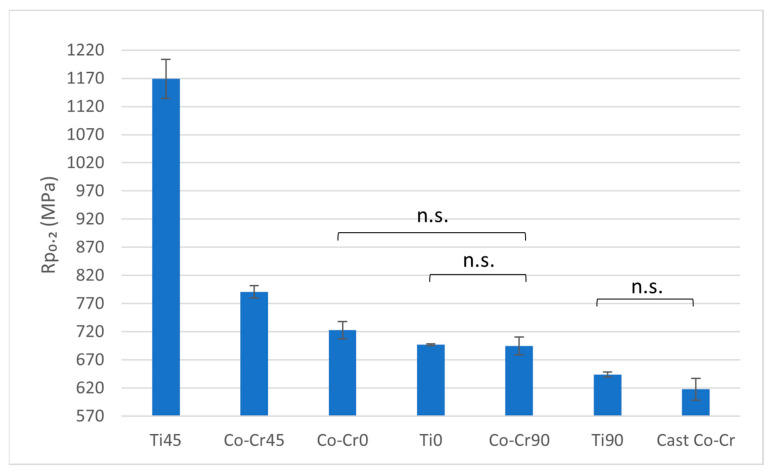
Rp_0.2_ ratio between tested materials. The graph shows the comparison of proof stress at 0.2% among cast Co-Cr alloy, 3D-printed Co-Cr alloy (Co-Cr0, Co-Cr45, Co-Cr90), and 3D-printed Titanflex® alloy (Ti0, Ti45, Ti90). Ti45 exhibited significantly higher yield strength compared to all other groups. Differences among marked groups were not statistically significant (n.s.), other differed significantly at *p* < 0.05. Error bars represent standard deviation.

Ti45 exhibits the highest 0.2% proof stress (Rp_0.2_) among all tested materials, significantly outperforming every other alloy. The other Titanflex® alloy samples (Ti0 and Ti90) show no statistically significant differences (n.s.) in Rp_0.2_ when compared to each other or to the Co-Cr alloy samples. Within the Co-Cr group, there are no statistically significant differences in Rp_0.2_ values between Co-Cr at 0° (Co-Cr0), Co-Cr45, and Co-Cr90. The cast Co-Cr alloy also shows no statistically significant difference in Rp_0.2_ compared to the Wironium variants, though its value is marginally lower (Figure 3 and Table 2).

**Figure 4:**
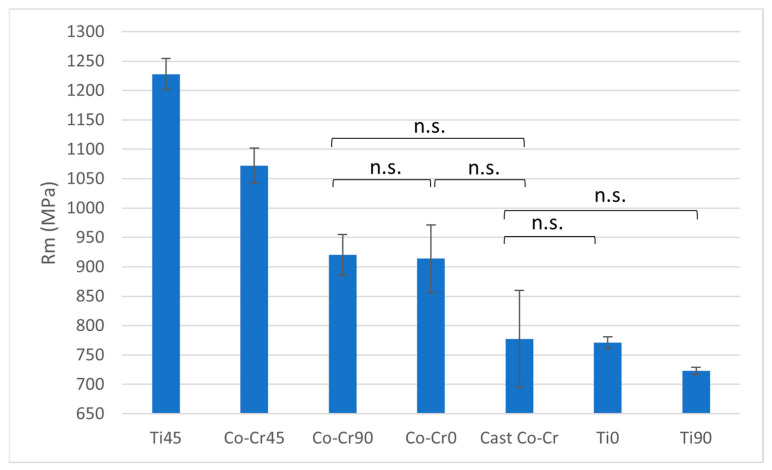
Rm ratio between tested materials. The graph shows the comparison of ultimate tensile strength among cast Co-Cr alloy, 3D-printed Co-Cr alloy (Co-Cr0, Co-Cr45, Co-Cr90), and 3D-printed Titanflex® alloy (Ti0, Ti45, Ti90). Ti45 showed the highest tensile strength. No statistically significant differences (n.s.) were observed among the marked groups, other differed significantly at *p* < 0.05. Error bars indicate standard deviation.

In terms of ultimate tensile strength (Rm), Ti45 demonstrates the highest values among all tested materials, significantly surpassing all others (*p* < 0.05). The other Titanflex® alloys (Ti0 and Ti90) show no statistically significant differences between each other or when compared to the Co-Cr alloys or cast Co-Cr. No statistically significant differences in Rm are observed within the Co-Cr alloy group (Figure 4 and Table 2). 

**Figure 5:**
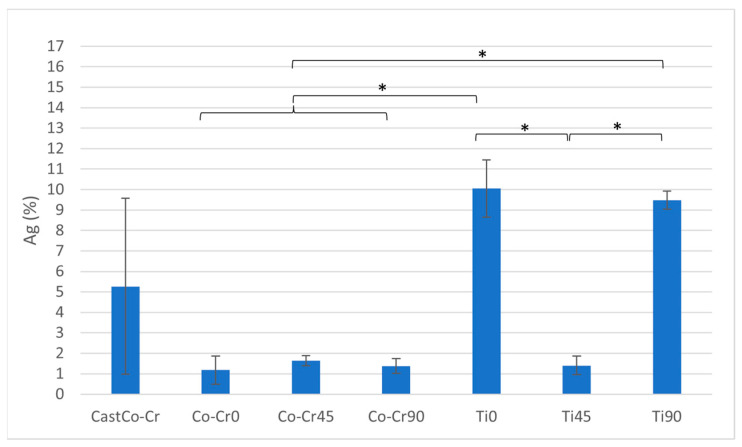
Ag ratio between tested materials. The graph shows the comparison of uniform elongation among cast Co-Cr alloy, 3D-printed Co-Cr alloy (Co-Cr0, Co-Cr45, Co-Cr90), and 3D-printed Titanflex® alloy (Ti0, Ti45, Ti90). Asterisks (*) indicate statistically significant differences (* *p* < 0.05). Error bars represent standard deviation.

Ti0 exhibits the highest uniform elongation (Ag), significantly surpassing all other materials (*p* < 0.05). Ti45 and Ti90 demonstrate lower Ag values compared to Ti0 but remain significantly higher than all other materials (*p* < 0.05). Statistically significant differences are observed among Ti0, Ti45, and Ti90. Among the Co-Cr alloys, no statistically significant differences in Ag are observed (Figure 5 and Table 3).

**Figure 6:**
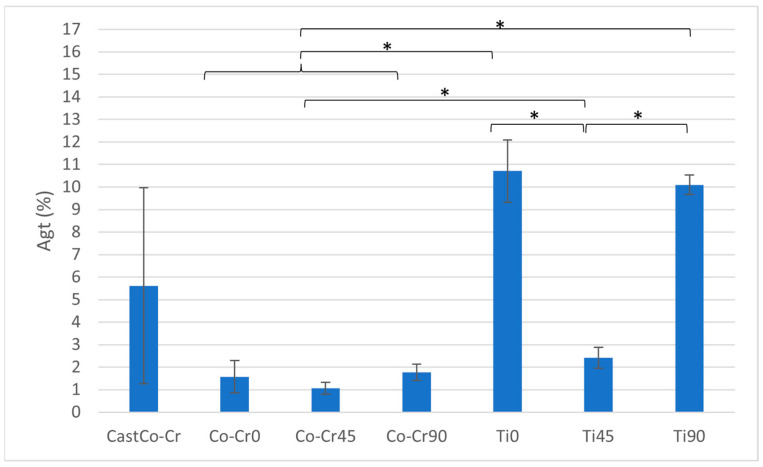
Total uniform elongation (Agt) ratio between tested materials. The graph shows the comparison of Agt values among cast Co-Cr alloy, 3D-printed Co-Cr alloy (Co-Cr0, Co-Cr45, Co-Cr90), and 3D-printed Titanflex® alloy (Ti0, Ti45, Ti90). Asterisks (*) indicate statistically significant differences (* *p* < 0.05). Error bars represent standard deviation.

For total uniform elongation (Agt), Ti0 demonstrates the highest value, significantly exceeding all other materials (*p* < 0.05). Ti90 also achieves high Agt values, which are significantly greater than Ti45, though both are statistically lower than Ti0 (*p* < 0.05). The Co-Cr alloys exhibit no statistically significant differences in Agt within their group, but show significantly lower values compared to all Titanflex® alloys (*p* < 0.05) (Figure 6 and Table 3).

**Figure 7:**
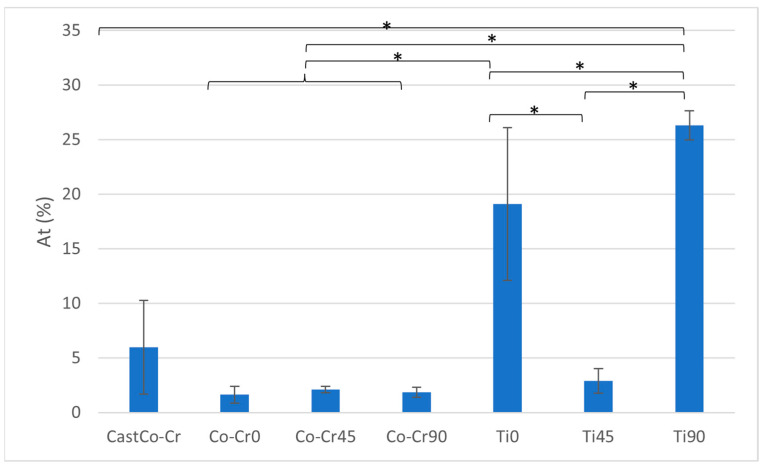
At ratio between tested materials. The graph shows the comparison of total breaking elongation among cast Co-Cr alloy, 3D-printed Co-Cr alloy (Co-Cr0, Co-Cr45, Co-Cr90), and 3D-printed Titanflex® alloy (Ti0, Ti45, Ti90). Asterisks (*) indicate statistically significant differences (* *p* < 0.05). Error bars represent standard deviation.

The cast Co-Cr alloy demonstrates a moderate total breaking elongation (At) compared to the other groups, with statistically significant differences observed when compared to Ti0 and Ti90 (*p* < 0.05). The printed Co-Cr alloys exhibit very low At values, with no significant differences observed among them. Ti0 achieves a significantly higher At compared to all printed Co-Cr and cast Co-Cr (*p* < 0.05), but it is significantly lower than Ti90 (*p* < 0.05). Ti45 demonstrates a slight increase in At compared to the Co-Cr groups but remains significantly lower than Ti0 and Ti90 (*p* < 0.05). Ti90 achieves the highest At value among all tested groups, showing statistically significant differences when compared to all other materials (*p* < 0.05) (Figure 7 and Table 3). 

**Figure 8:**
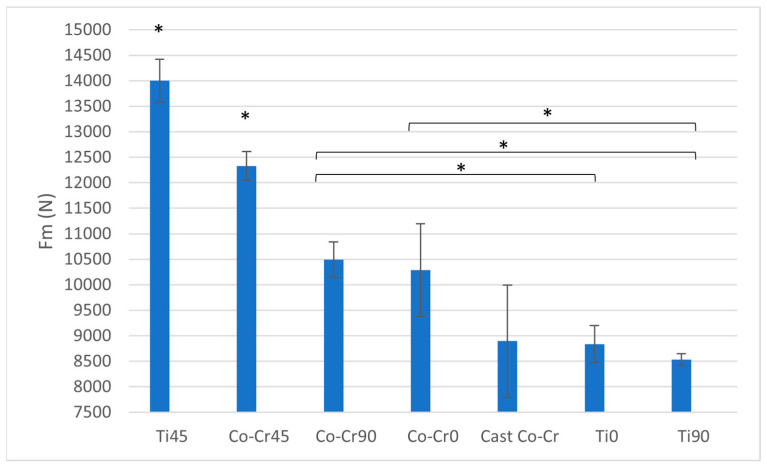
Fm ratio between tested materials. The graph shows the comparison of maximum load among cast Co-Cr alloy, 3D-printed Co-Cr alloy (Co-Cr0, Co-Cr45, Co-Cr90), and 3D-printed Titanflex® alloy (Ti0, Ti45, Ti90). Asterisks (*) indicate statistically significant differences (* *p* < 0.05). Error bars represent standard deviation.

Regarding maximum load (Fm), Ti45 exhibits the highest value among all tested materials, significantly surpassing all others (*p* < 0.05). Co-Cr45 shows a slightly lower Fm than Ti45 but remains significantly higher than Wironium0, Wironium90, and cast Co-Cr (*p* < 0.05). Co-Cr90 achieves an intermediate Fm value, which is significantly higher than Ti0, Ti90, and cast Co-Cr (*p* < 0.05), but lower than Ti45 and Co-Cr45 (*p* < 0.05). Co-Cr0 demonstrates a similar Fm to Wironium90, with no significant difference between the two, though both are significantly higher than Ti0, Ti90, and cast Co-Cr (*p* < 0.05). The cast Co-Cr alloy, Ti0, and Ti90 show significantly lower Fm values compared to Co-Cr45 variants and Ti45 (*p* < 0.05), with no statistically significant differences observed among these three groups (Figure 8 and Table 3). 

## 4. Discussion

The mechanical properties of Titanflex® and Co-Cr alloys were evaluated to assess their potential as materials for metal base of removable dentures. Titanflex® alloys demonstrated a significantly lower modulus of elasticity (E) compared to Co-Cr alloys, with Ti90 exhibiting the lowest stiffness among all materials, indicating enhanced flexibility. Co-Cr alloys, on the other hand, displayed higher E values, with orientation-dependent variations, such as lower E in Co-Cr90 compared to Co-Cr45, highlighting anisotropic mechanical behavior. These findings align with previous research indicating that titanium alloys exhibit low elastic moduli, often tailored to match biological structures like bones. These alloys typically display moduli in the range of 17–65 GPa, which is significantly lower than that of Co-Cr alloys [23,24]. This lower modulus allows for greater flexibility, which may be beneficial for reducing stresses on the underlying soft tissues (e.g., the gums, and periodontal ligaments of the supporting teeth) and making the denture more comfortable but may be less suitable for larger partial dentures that need to withstand greater mechanical stresses, whereas Co-Cr alloys are traditionally valued for their rigidity and dimensional stability. Future research should address those concerns.

The proof stress (Rp_0.2_) of Ti45 emerged as the highest among all materials, surpassing both other Titanflex® variants and Co-Cr alloys, suggesting excellent resistance to plastic deformation. This high proof stress is important for ensuring the denture’s structural integrity under normal chewing forces without permanent deformation. A higher Rp_0.2_ ensures that the denture will maintain its shape and functional performance over time. However, Ti0 and Ti90 exhibited Rp_0.2_ values similar to those of Co-Cr alloys, which demonstrated uniform performance across different printing orientations. This divergence from some earlier findings, where Co-Cr alloys consistently outperformed titanium-based systems in terms of yield strength, may be attributed to differences in manufacturing parameters, printing orientation, and alloy composition. Such variability highlights the importance of process optimization in additive manufacturing.

Ultimate tensile strength (Rm) followed a similar pattern, with Ti45 outperforming all other alloys, while Ti0, Ti90, and the Co-Cr group showed no significant differences. The high Rm of Ti45 reinforces its suitability for applications requiring resistance to fracture under stress, paralleling established findings that titanium alloys provide reliable strength under mechanical loading. In earlier studies of dental alloys cobalt–chromium alloys typically had higher tensile strengths, ranging from approximately 1100 MPa to over 1500 MPa [24,25]. Our findings therefore partially contrast with the established literature, suggesting that the additive manufacturing process can endow titanium alloys with mechanical advantages that challenge conventional assumptions about Co-Cr superiority. This points to the need for careful comparative studies between conventionally cast and additively manufactured alloys.

In terms of ductility, Titanflex® alloys displayed markedly superior performance compared to Co-Cr alloys. Ti0 exhibited the highest elongation at break (Ag), total uniform elongation (Agt), and breaking elongation (At), followed by Ti90 and Ti45, all of which significantly outperformed Co-Cr alloys. Titanium alloys generally exhibit higher ductility due to their ability to undergo significant plastic deformation before failure. For example, the Ti-6Al-7Nb alloy demonstrates superior torsional ductility compared to Co-Cr alloys, maintaining high torsional strength even after laser welding. This substantial ductility supports the hypothesis that titanium alloys can better accommodate deformation under functional loading, potentially reducing the risk of fracture. In contrast, Co-Cr alloys, while exhibiting high strength and wear resistance, are typically less ductile. For instance, Co-Cr dental castings show brittle fracture behavior under torsional stress, indicating limited capacity for plastic deformation [17]. Such contrasting behavior emphasizes a clinical trade-off: while titanium alloys may improve patient comfort by better absorbing functional stresses, Co-Cr alloys provide reassurance in terms of rigidity and long-standing clinical reliability. This duality underscores the importance of tailoring material choice to case-specific requirements.

The maximum force (Fm) results further underscore the superior load-bearing capacity of Ti45, which recorded the highest Fm, followed by Co-Cr45. On the other hand, the Ti0, Ti90, and cast Co-Cr displayed significantly lower Fm values, suggesting limitations in their load-bearing performance relative to Ti45 and Co-Cr45. In previous studies, Co-Cr alloys generally exhibited higher strength than titanium alloys, with maximum tensile forces often correlating to their tensile strength of approximately 1100 MPa and above. This makes them suitable for applications requiring high wear resistance and load-bearing capabilities [26]. In studies of prosthetic applications, Co-Cr frameworks withstood higher forces before failure compared to CP (Commercially Pure) titanium frameworks, underlining their superior force resistance [27]. The superior performance of Ti45 observed here could be linked to microstructural features induced by the printing process; however, because this study did not include detailed fractographic or surface microstructural analysis, this remains speculative. Including such analyses in future studies would enable more definitive explanations for the observed discrepancies.

Overall, this discussion demonstrates that while our results are broadly consistent with previous research, certain divergences (e.g., the unexpectedly high proof stress and tensile strength of Ti45) suggest that additive manufacturing may fundamentally alter the performance hierarchy between titanium and Co-Cr alloys. Critical reflection on these divergences points to process-dependent phenomena as a likely explanation. Future work should therefore integrate dynamic testing, surface quality assessment, and finite element modeling to simulate intraoral loading conditions. Such approaches would not only validate the current findings, but also deepen the understanding of how microstructure, surface finish, and anisotropy influence clinical performance.

## 5. Conclusions

Findings from this study collectively support the hypothesis that titanium-based alloys, particularly Ti45, offer a compelling combination of strength, flexibility, and ductility, positioning them as promising candidates for removable denture bases. The superior mechanical performance of Ti45—especially its high proof stress, tensile strength, and balanced elongation properties—indicates its potential to withstand functional stresses while enhancing patient comfort. In contrast, Co-Cr alloys, while offering higher rigidity and established clinical reliability, may be less adaptable to the dynamic loading conditions and biological environment of the oral cavity.

The most significant contribution of this study is that Titanflex®, as a titanium-based alloy, has not previously been systematically investigated for dental applications, making these findings a novel addition to the existing body of knowledge. Furthermore, the role of 3D printing orientation in shaping mechanical properties is emphasized, offering new insights for optimizing denture base frameworks produced by additive manufacturing.

Nonetheless, some limitations should be acknowledged. The present work did not include a detailed examination of the surface microstructure or fracture sites, which could have provided additional insight into the underlying failure mechanisms. Likewise, the focus was restricted to static mechanical testing, without incorporating dynamic or fatigue analyses that better reflect intraoral conditions.

Future studies should therefore expand this research by conducting dynamic testing on the same alloys, integrating surface quality parameters into the interpretation of mechanical results, and applying finite element modeling to simulate the performance of 3D-printed Titanflex dentures under realistic oral conditions. Such investigations would strengthen the clinical relevance of the findings and provide a more comprehensive understanding of the alloy’s behavior in functional environments.

By acknowledging these limitations and outlining directions for future research, this study establishes a strong foundation for the further development and clinical integration of 3D-printed titanium denture base frameworks.

## Figures and Tables

**Table 1:** Geometrical and elastic properties of Co-Cr and Ti materials. This table displays the initial cross-sectional area (S_0_) and E-Modulus (E) for cast and 3D-printed cobalt–chromium (Co-Cr) and titanium (Ti) samples. The data includes the mean (M) and standard deviation (SD) for each parameter.

Material	Angle (°)	S_0_ (mm^2^)	SD	E (GPa)	SD
Cast Co-Cr	–	11.42	0.284	232.4	41.01
Co-Cr	0	11.24	0.146	238.0	8.883
Co-Cr	45	11.50	0.092	250.9	3.372
Co-Cr	90	11.40	0.120	238.3	3.470
Ti	0	11.46	0.334	117.6	2.663
Ti	45	11.40	0.104	121.1	2.542
Ti	90	11.80	0.197	114.3	1.137

**Table 2:** Strength properties of Co-Cr and Ti materials. This table includes proof stress at 0.2% (Rp_0.2_) and ultimate tensile strength (Rm). Each value is given with mean (M) and standard deviation (SD).

Material	Angle (°)	Rp_0.2_ (MPa)	SD	Rm (MPa)	SD
Cast Co-Cr	–	617.7	19.23	7776	82.4
Co-Cr	0	722.6	15.41	913.9	57.74
Co-Cr	45	791.1	10.71	1072	29.77
Co-Cr	90	694.5	15.77	920.3	34.76
Ti	0	697.2	1.563	771	9.695
Ti	45	1169	34.5	1228	26.23
Ti	90	644	4.743	723	6.403

**Table 3:** Ductility and load-bearing properties of Co-Cr and Ti materials. This table covers uniform elongation (Ag), total uniform elongation (Agt), breaking elongation (A), total breaking elongation (At), and the maximum load (Fm) each sample withstood. The data includes the mean (M) and standard deviation (SD) for each parameter.

Material	Angle (°)	Ag (%)	SD	Agt (%)	SD	A (%)	SD	At (%)	SD	Fm (N)	SD
Cast Co-Cr	–	5.269	4.296	5.612	4.359	5.668	4.217	5.982	4.301	8894	1099
Co-Cr	0	1.183	0.69	1.568	0.719	1.262	0.761	1.636	0.781	10,285	910.5
Co-Cr	45	1.635	0.246	1.062	0.263	1.666	0.272	2.091	0.287	12,331	278.3
Co-Cr	90	1.38	0.356	1.766	0.362	1.462	0.452	1.844	0.454	10,491	348.3
Ti	0	10.05	1.391	10.71	1.383	18.59	7.021	19.09	6.999	8840	362.7
Ti	45	1.406	0.453	2.419	0.468	1.897	1.113	2.904	1.117	13,999	421.2
Ti	90	9.473	0.441	10.1	0.441	25.84	1.33	26.31	1.335	8533	117.3

## Data Availability

The original contributions presented in the study are included in the article, further inquiries can be directed to the corresponding author.

## Abbreviations

3D	Three-Dimensional
Ag	Uniform Elongation
Agt	Total Uniform Elongation
A	Breaking Elongation
At	Total Breaking Elongation
CLP	Classification, Labeling and Packaging (Regulation (EC) No. 1272/2008)
CMR	Carcinogenic, Mutagenic, and Toxic to Reproduction
Co	Cobalt
Co-Cr	Cobalt-Chromium
CNC	Computer Numerical Control
Cr	Chromium
d_0_	Initial Diameter of Gauge Length
d_1_	Diameter of Gripping Section
E	Modulus of Elasticity
EU	European Union
Fm	Maximum Load
h_min_	Minimum Height of Gripping Section
ISO	International Organization for Standardization
L_0_	Gauge Length
Lc_min_	Minimum Clamping Length
Lt_min_	Minimum Total Length
MDR	Medical Device Regulation (EU 2017/745)
R_min_	Minimum Radius Between Gauge and Grip Section
Rm	Ultimate Tensile Strength
Rp_0.2_	Proof Stress at 0.2% Strain
S_0_	Initial Cross-Sectional Area
SD	Standard Deviation
SLM	Selective Laser Melting
Ti	Titanium
Ti0	Titanflex® Printed at 0°
Ti45	Titanflex® Printed at 45°
Ti90	Titanflex® Printed at 90°
